# Sarcomatoid cutaneous squamous cell carcinoma of the heel: a rare localization of an aggressive variant of cutaneous squamous cell carcinoma

**DOI:** 10.1093/omcr/omag056

**Published:** 2026-05-10

**Authors:** Line Farhat, Ouiame Eljouari, Salma Fahmi, Salim Gallouj

**Affiliations:** Department of Dermatology and Venereology, Mohamed VI University Hospital Centre, Faculty of Medicine and Pharmacy of Tangier (FMPT), Abdelmalek Essaâdi University, La Nouvelle Ville Ibn Battouta, Tangier 90000, Morocco; Department of Dermatology and Venereology, Mohamed VI University Hospital Centre, Faculty of Medicine and Pharmacy of Tangier (FMPT), Abdelmalek Essaâdi University, La Nouvelle Ville Ibn Battouta, Tangier 90000, Morocco; Department of Dermatology and Venereology, Mohamed VI University Hospital Centre, Faculty of Medicine and Pharmacy of Tangier (FMPT), Abdelmalek Essaâdi University, La Nouvelle Ville Ibn Battouta, Tangier 90000, Morocco; Department of Dermatology and Venereology, Mohamed VI University Hospital Centre, Faculty of Medicine and Pharmacy of Tangier (FMPT), Abdelmalek Essaâdi University, La Nouvelle Ville Ibn Battouta, Tangier 90000, Morocco

**Keywords:** cutaneous squamous cell carcinoma, spindle cell tumour, heel tumour, immunohistochemistry, epithelial–mesenchymal transition

## Abstract

Sarcomatoid cutaneous squamous cell carcinoma (SCSCC) is a rare and aggressive variant of squamous cell carcinoma characterized by spindle-cell morphology resulting from epithelial–mesenchymal transition rather than true biphasic differentiation. Its sarcomatoid component may mimic other spindle-cell neoplasms, making diagnosis challenging. We report a 63-year-old chronic smoker presenting with a giant, rapidly enlarging ulcerated tumour of the left heel, initially misdiagnosed as a callus and later as Kaposi sarcoma and melanoma. Imaging showed no metastasis. The patient underwent wide trans-calcaneal resection with prophylactic inguinal lymph-node dissection. Histology demonstrated a high-grade spindle-cell malignancy expressing epithelial markers (CK5/6, AE1/AE3, p63) with negativity for specific melanocytic markers, confirming SCSCC (pT4N0, AJCC 8th edition). After 3 months of follow-up, no recurrence or metastasis was observed. This case highlights diagnostic pitfalls and the importance of comprehensive immunohistochemistry and multidisciplinary management.

## Introduction

Sarcomatoid cutaneous squamous cell carcinoma (SCSCC) is an uncommon and aggressive variant of conventional cutaneous squamous cell carcinoma [1–3], defined by a monophasic epithelial malignancy exhibiting sarcomatoid spindle-cell morphology resulting from epithelial–mesenchymal transition [3–5]. Because the spindle-cell component may closely resemble other cutaneous spindle-cell neoplasms, including desmoplastic melanoma and pleomorphic dermal sarcoma, accurate diagnosis often requires an extended immunohistochemical panel [5–7].

Primary cutaneous SCSCC remains rare, with only limited cases reported in the literature [3].

## Case report

A 63-year-old man, a chronic smoker, was referred for evaluation of a giant ulcerated exophytic tumour of the left heel evolving over three years. Initially considered a simple callus, the lesion rapidly increased in size, became painful and oozing, and interfered with walking.

An external biopsy suggested Kaposi sarcoma. Subsequent immunohistochemical analysis at another institution favoured melanoma, prompting referral to our centre.

**Figure 1:**
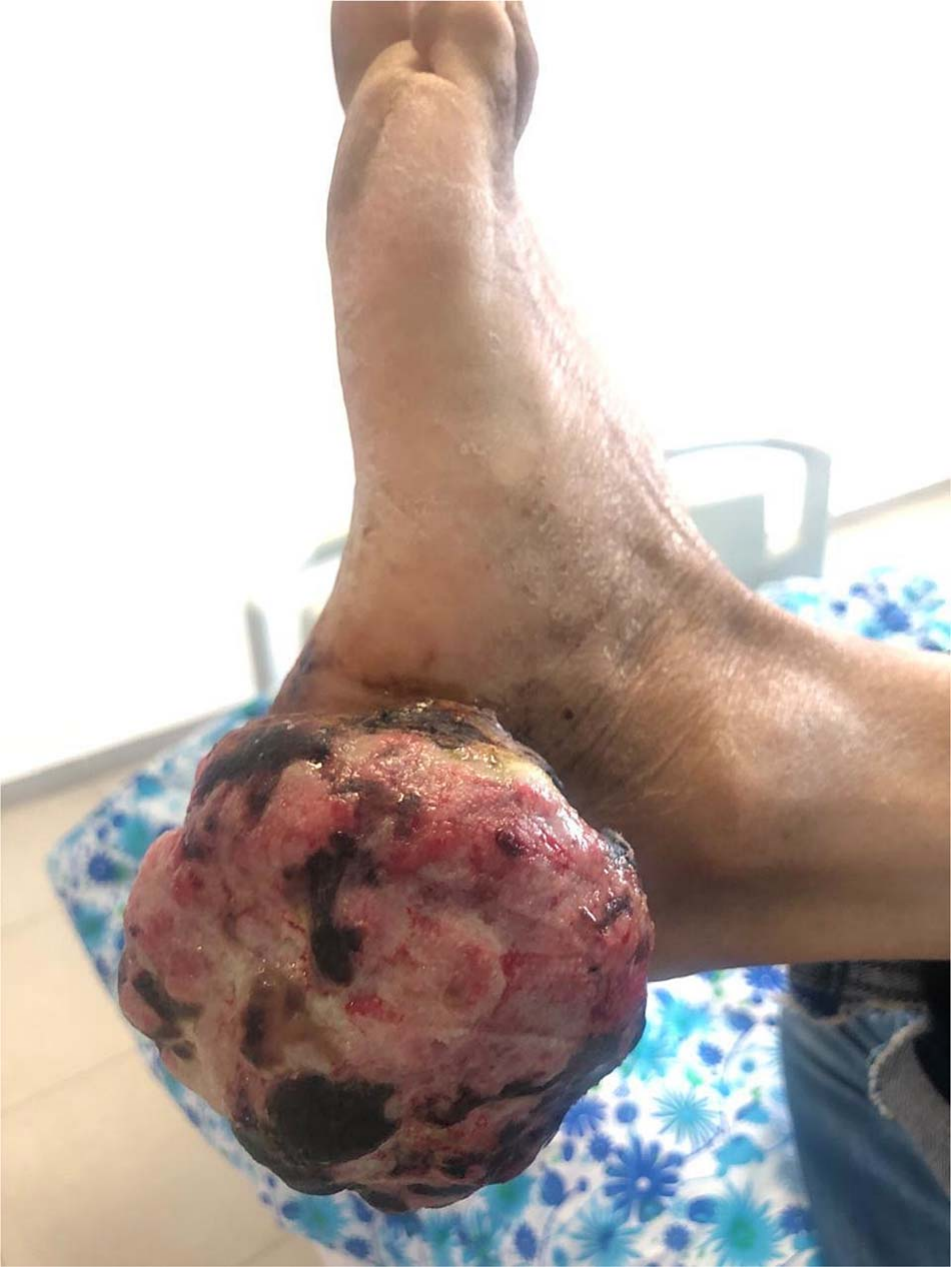
Lateral view of the large ulcerated exophytic tumour of the left heel.

**Figure 2:**
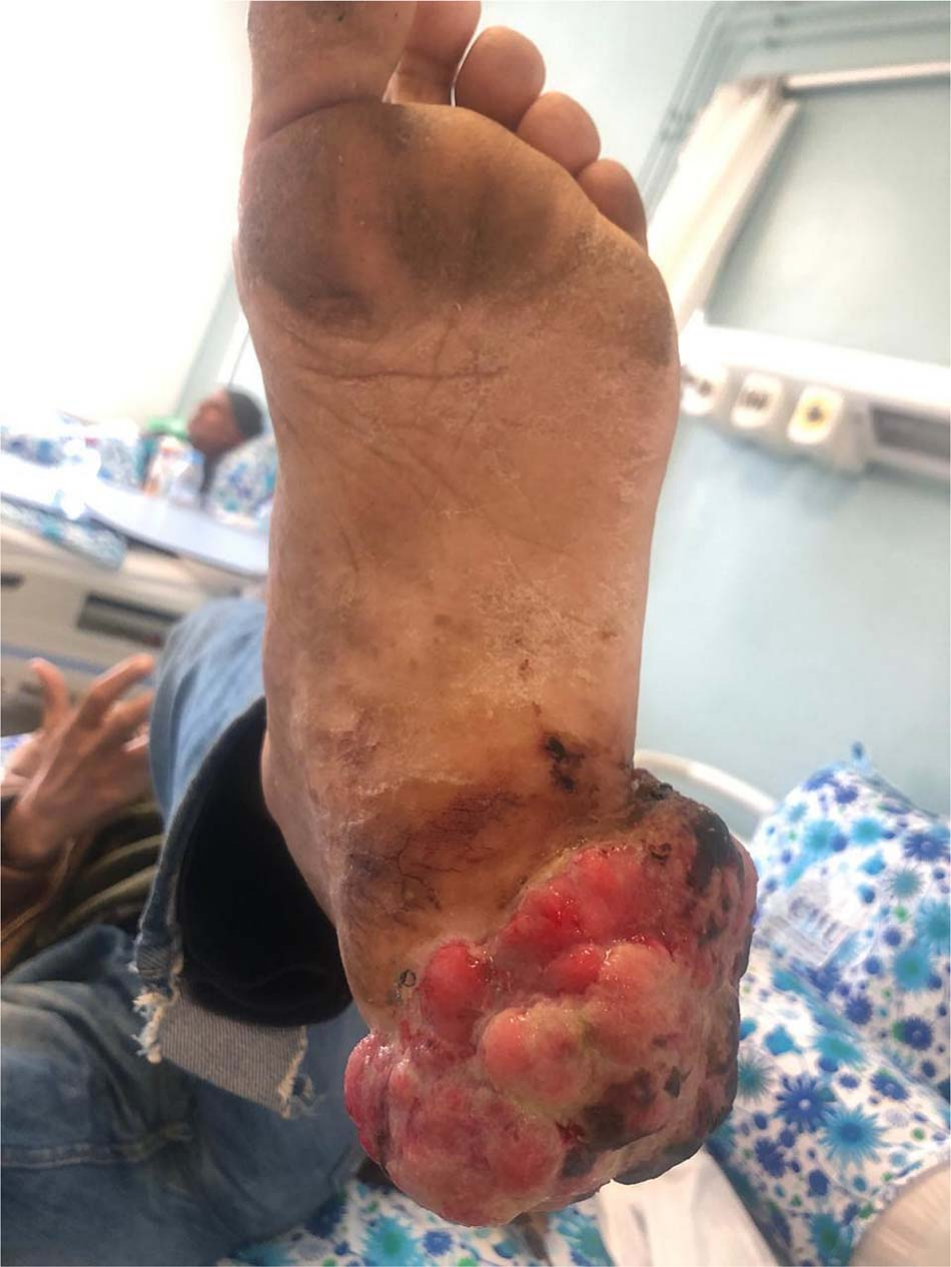
Frontal view of the tumour showing extensive necrotic and bleeding areas.

On examination, the patient was in good general condition. Dermatological examination found a large exophytic, ulcerated, spontaneously bleeding tumour of the left heel, measuring approximately 6 cm, with surface necrosis and functional gait impairment (Figs 1 and 2). No palpable lymphadenopathy was detected.

Baseline laboratory tests were unremarkable. Contrast-enhanced CT of the chest, abdomen and pelvis showed no distant metastases.

A wide trans-calcaneal excision with safety margins and inguinal lymph node dissection in the Scarpa triangle was performed.

**Figure 3:**
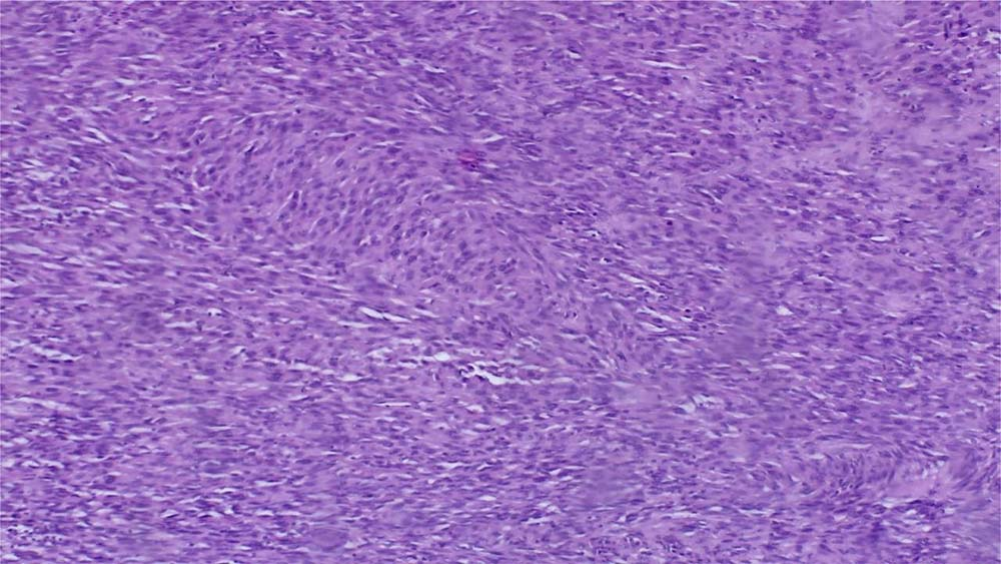
Histopathological section (H&E, ×20) showing malignant spindle-cell proliferation with marked atypia.

Histopathology revealed a malignant spindle cell proliferation with an exophytic budding architecture (Fig. 3), composed of ovoid to spindle-shaped cells showing moderate-to-severe atypia, a high mitotic index (16 mitoses/10 HPF), focal tumour necrosis and scant stroma. Margins were free of tumour, with 15 mm clearance to bone and 18 mm to soft tissue.

All 17 dissected lymph nodes were tumour-free (17 N–/17 N). According to AJCC 8th edition, staging was pT4N0Mx.

**Figure 4:**
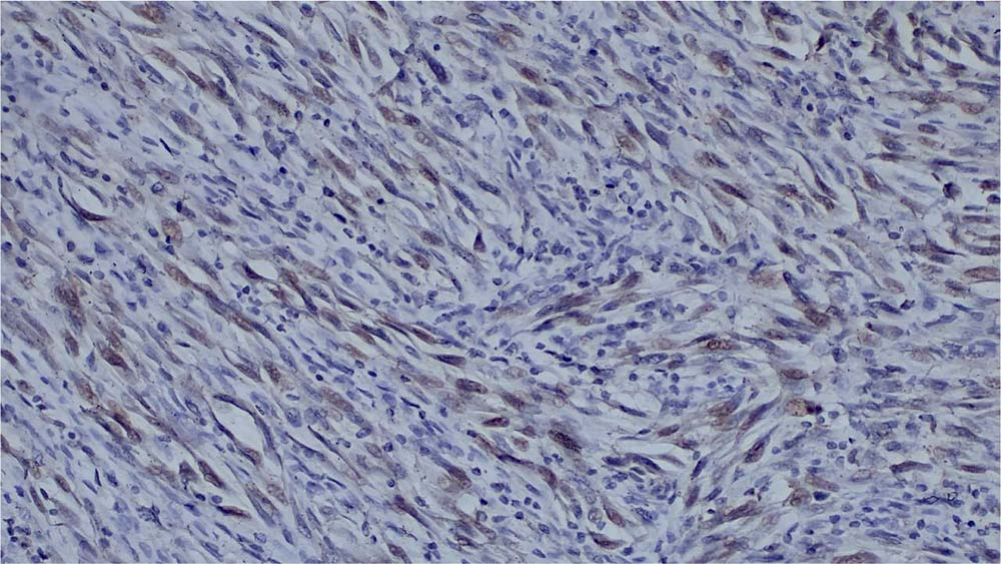
Immunohistochemical staining showing strong CK5/6 positivity.

**Figure 5:**
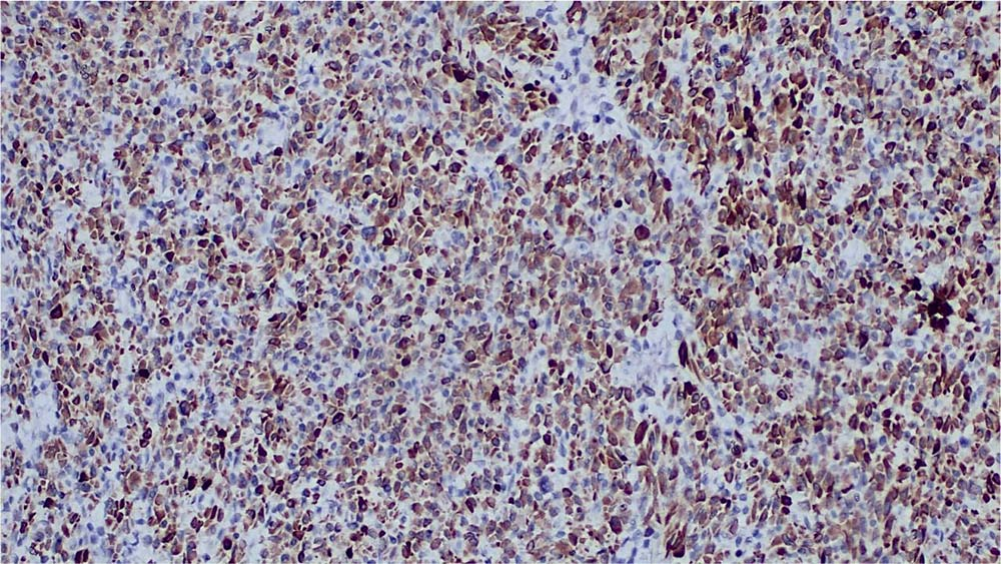
Immunohistochemical staining showing diffuse AE1/AE3 expression.

**Figure 6:**
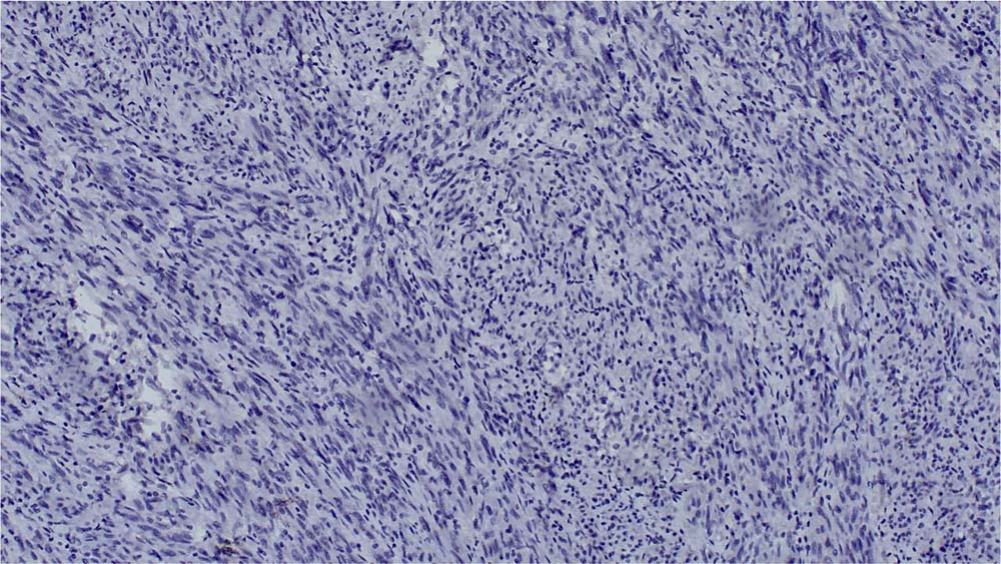
Negative melan-a staining, ruling out melanocytic differentiation.

Immunohistochemistry showed diffuse expression of epithelial markers (CK5/6, AE1/AE3, p63) and focal, non-specific S100 expression (Figs 4 and 5), with negativity for melanocytic markers (Fig. 6). The overall profile supported a diagnosis of sarcomatoid cutaneous squamous cell carcinoma of the heel.

Reconstructive heel surgery was performed. After 3 months of postoperative follow-up, the patient remained free of local recurrence and distant metastasis.

## Discussion

Sarcomatoid squamous cell carcinoma is a rare and aggressive variant of squamous cell carcinoma, most commonly arising in visceral sites such as the lung, upper aerodigestive tract, breast, kidney, bladder and thyroid, whereas primary cutaneous involvement is uncommon [2, 8]. Its morphology typically reflects a monophasic epithelial malignancy with sarcomatoid spindle-cell features resulting from epithelial–mesenchymal transition. Various histological configurations have been described, including associations with basaloid carcinoma or true mesenchymal sarcoma [8].

The most accepted hypothesis suggests a monoclonal epithelial origin, with the spindle cell component deriving through epithelial–mesenchymal transition [4, 9]. This is supported by the frequent expression of epithelial markers within the spindle component and by molecular evidence of clonal relatedness [5, 9, 10].

Diagnosis is challenging because SCSCC may mimic desmoplastic melanoma or undifferentiated pleomorphic sarcoma [1, 7], as illustrated by the misdiagnoses in our case. A deep and representative biopsy with an extended immunohistochemical panel is essential.

Spindle-cell tumours of the skin represent a well-recognized diagnostic challenge and require a broad immunohistochemical panel. The typical immunoprofile of SCSCC includes positivity for cytokeratins (AE1/AE3, CK5/6) and squamous markers (p63 or p40), confirming epithelial lineage despite sarcomatoid morphology, with negativity for specific melanocytic markers such as SOX10 and Melan-A. This approach is essential to distinguish SCSCC from desmoplastic melanoma, atypical fibroxanthoma, pleomorphic dermal sarcoma and true cutaneous carcinosarcoma.

Management is based on wide surgical excision with free margins. Lymph node dissection is indicated when lymphadenopathy is present or in selected high-risk tumours [1]. Adjuvant radiotherapy may be considered in locally advanced, recurrent or high-risk cases, particularly in the presence of perineural invasion, positive margins or nodal involvement [3].

In the present case, inguinal lymph-node dissection was performed despite the absence of clinically palpable lymphadenopathy because the tumour displayed multiple high-risk features, including large size, deep invasion (pT4) and aggressive sarcomatoid histology. The decision was validated in a multidisciplinary tumour board setting in view of the known metastatic potential of high-risk cutaneous SCC variants. Adjuvant radiotherapy was discussed but was not retained given the absence of nodal involvement and the achievement of complete surgical excision with clear margins.

This case is remarkable due to its exceptional heel localisation and prolonged misinterpretation as benign or alternative malignancies. It highlights the importance of early biopsy and multidisciplinary evaluation of atypical heel lesions.

However, the follow-up was limited to three months, after which the patient was lost to follow-up, which constitutes a limitation in assessing the long-term outcome, especially considering the aggressive behavior of sarcomatoid cutaneous squamous cell carcinoma.
